# Surface Glucan Structures in *Aeromonas* spp.

**DOI:** 10.3390/md19110649

**Published:** 2021-11-22

**Authors:** Elena Mendoza-Barberá, Susana Merino, Juan Tomás

**Affiliations:** 1 Department of Genetics, Microbiology and Statistics, University of Barcelona, 08028 Barcelona, Spain; smerino@ub.edu (S.M.); jtomas@ub.edu (J.T.); 2 Institut de Recerca en Nutrició i Seguretat Alimentària (INSA), 08921 Santa Coloma de Gramenet, Spain

**Keywords:** *Aeromonas*, capsule polysaccharide, O-antigen, LPS, α-glucan, glycosylation

## Abstract

*Aeromonas* spp. are generally found in aquatic environments, although they have also been isolated from both fresh and processed food. These Gram-negative, rod-shaped bacteria are mostly infective to poikilothermic animals, although they are also considered opportunistic pathogens of both aquatic and terrestrial homeotherms, and some species have been associated with gastrointestinal and extraintestinal septicemic infections in humans. Among the different pathogenic factors associated with virulence, several cell-surface glucans have been shown to contribute to colonization and survival of *Aeromonas* pathogenic strains, in different hosts. Lipopolysaccharide (LPS), capsule and α-glucan structures, for instance, have been shown to play important roles in bacterial–host interactions related to pathogenesis, such as adherence, biofilm formation, or immune evasion. In addition, glycosylation of both polar and lateral flagella has been shown to be mandatory for flagella production and motility in different *Aeromonas* strains, and has also been associated with increased bacterial adhesion, biofilm formation, and induction of the host proinflammatory response. The main aspects of these structures are covered in this review.

## 1. Introduction

The *Aeromonadaceae* family comprises Gram-negative, facultative anaerobic, and chemoorganotrophic bacteria, with an optimal growing temperature of 22–37 °C [1]. Members of this family are generally motile by polar flagellation, and are able to reduce nitrates to nitrites. They also have the ability to catabolize glucose and other carbohydrates, producing acids and often gases. In particular, the genus *Aeromonas* is comprised of water-borne bacteria, ubiquitously found in aquatic environments (e.g., rivers, lakes, ponds, seawater estuaries, drinking water, groundwater or wastewater), and also identified in different types of food (e.g., dairy products, vegetables, meats, poultry, shellfish and fish) [1,2]. Generally, the members of this genus are classified in two different groups: the non-motile, psychrophilic species (e.g., *A. salmonicida*) and the motile, mesophilic species (e.g., *A. hydrophila*, *A. caviae*, and *A. sobria*) [2]. *Aeromonas* spp. could potentially pose a serious risk to public health, as many strains are able to grow and produce exotoxins at low temperatures and high salt concentrations [3,4]. In fact, they are emerging as the causative agents of gastrointestinal and extraintestinal diseases in a wide range of animals [2]. In fish, for instance, *A. salmonicida* induces systemic furunculosis in *Salmonidae*, and several mesophilic species (e.g., *A. hydrophila*, *A. jandaei* and *A. veronii*) have been found responsible for hemorrhagic septicemia in this and other fish [5,6,7], causing major economic losses in aquaculture [8,9]. In humans, they are implicated in several intestinal and extraintestinal septicemic infections, ranging from relatively mild illnesses (e.g., acute gastroenteritis or superficial wound infections) to more complicated pathologies (e.g., respiratory-tract or eye infections), or even life-threatening conditions (e.g., septicemia, meningitis, myonecrosis or osteomyelitis) [10,11]. The enteropathogenic role of *Aeromonas* spp. has been reported in several publications, entailing a high risk at even moderate or low concentrations [11,12,13]. Different outbreaks related to *Aeromonas* spp. have in fact been reported in the last few decades [14,15,16,17], and it was also the most isolated microorganism following natural disasters such as the 2005 Hurricane Katrina in the United States and the 2001 tsunami in Thailand [18,19]. Moreover, highlighting the potential risk of these bacteria for human health, antimicrobial resistant *Aeromonas* strains have been found in both domestic and wild animals [20,21], and clinical strains isolated from extraintestinal infections have been shown to be resistant to several antibiotics [22]. The number of reported *Aeromonas* spp. infections in humans has been steadily rising in recent years, presenting a serious threat to the increasing population of immunocompromised patients [13,23], and will remain a human health problem in the near future, considering the increased and more susceptible population of elderly people with potential underlying diseases [12].

Among the different pathogenic factors associated with virulence of *Aeromonas* spp., several cell-surface glucans have been reported to play important roles in host–pathogen interactions, contributing to adherence, colonization, and overall survival of pathogenic strains in different hosts [24]. For instance, several mesophilic *Aeromonas* spp. have been reported to have glycosylated flagella, and these sugar modifications have been shown to be involved in several pathogenic processes, including motility, biofilm formation, adhesion, and stimulation of the immune response [25,26,27,28,29]. In addition, the lipopolysaccharide (LPS) molecule, a surface glycoconjugate exclusively found in Gram-negative bacteria, has been widely reported to be a key elicitor of host innate immune responses through TLR4-mediated signaling [30], sometimes provoking an overstimulated response that leads to host multiorgan injury and dysfunction [31]. Capsular polysaccharides (CPSs), found on the cell surface of a broad range of bacterial species, also seem to have a role in the virulence and colonization potential of both motile (*A. piscicola* AH-3, previously known as *A. hydrophila*) and non-motile (*A. salmonicida*) *Aeromonas* spp. [32,33], and α-glucan, a surface polysaccharide widely found in nature, has been shown to be crucial for biofilm formation in *A. hydrophila* AH-1 and *A. piscicola* AH-3 [34].

The main structural and molecular aspects of the surface glucans identified in *Aeromonas* spp. to date, as well as their biological implications, are discussed in this review.

## 2. Glycosylated Flagella

**Figure 1:**
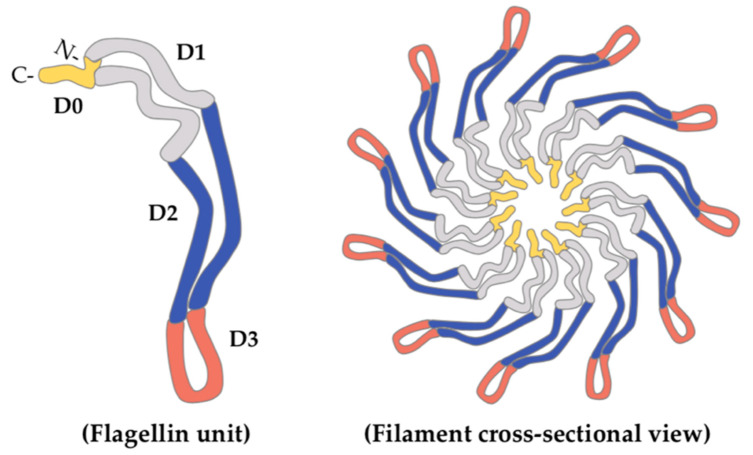
Schematic representation of flagellin structure and arrangement into the flagellar filament. Flagellins are arranged into 11 protofilaments in a way that domains D0 (yellow) and D1 (grey) remain in the interior of the flagellar filament, while domains D2 (blue) and D3 (red) are exposed to the outer surface.

Bacterial flagella are protein structures whose main function is motility, both in liquid and solid environments. Mesophilic *Aeromonas* spp. constitutively express a single polar flagellum for movement in liquid environments, and 50–60% of clinical isolates also possess an additional inducible lateral-flagella system, expressed for motility in solid or viscous conditions [35]. Structurally, the flagellar system is comprised of the basal body, embedded in the bacterial surface, and the hook and the filament, constituting the external part. The flagellar filament is in turn composed of flagellin proteins, assembled into 11 protofilaments [36], whose expression differs in number and diversity among different Gram-negative species. For instance, while *Vibrio parahaemolyticus* is able to synthesize six different polar flagellins [37], *Escherichia coli* and *Salmonella* *enterica* serovar Typhimurium express many copies of a single one [38]. In this regard, mesophilic *Aeromonas* spp. have been shown to generally express two polar (FlaA and FlaB) and one lateral (LafA) flagellin [39,40], although a few strains (e.g., *A. caviae* Sch3N) express two different flagellin proteins (LafA1 and LafA2) in their lateral-flagella filaments [41]. Structurally, as determined in *S. enterica* serovar Typhimurium, functional flagellins are comprised of four domains: D0, D1, D2 and D3 (Figure 1) [42]. Domains D0 and D1 present an α-helix conformation, and are comprised by the well-conserved N- and C-terminal ends of the flagellin protein. These domains are embedded in the inner core of the filament, and are necessary for filament architecture and motility functions. On the other hand, the central domains D2 and D3 present a variable β-sheet conformation, and are exposed to the outer surface. This is of particular importance because this D2/D3 region can be subjected to glycosylation, as observed in *A. caviae* [43] and other Gram-negative species (e.g., *Helicobacter pylori* and *Campylobacter jejuni* [44,45]), and seems to be involved in the adhesin-like behavior of flagella in *A. piscicola* [28].

Although protein glycosylation was long thought to exist only in eukaryotes, it has been shown to be essential for a multitude of cellular functions in prokaryotes as well [46]. In Gram-negative bacteria, several surface-associated glycoproteins have been described. Some examples include the pilins of *Pseudomonas aeruginosa* and *Neisseria* spp.; the adhesins HMW1 (*Haemophilus influenzae*), TibA and AIDA-1 (*E. coli*); and the flagellins of *P. aeruginosa*, *H. pylori*, *Clostridium botulinum*, and *C. jejuni/C. coli* [47]. Flagellin glycosylation, in particular, has been shown to have different biological functions, depending on the species. For instance, in *C. jejuni* and *H. pylori*, glycosylation is necessary for filament assembly [44,48], and plays an essential role in the intestine colonization process [49,50]. On the contrary, in *P. aeruginosa*, flagellin glycosylation seems to be dispensable for both flagellar assembly and motility, but it is needed for the proinflammatory action of this protein [51,52]. In *Aeromonas* spp., glycosylation of both polar and lateral flagella has been shown to be required for flagellar assembly and stability, in *A. caviae* and *A. piscicola* AH-3 [25,26]. Polar-flagella glycosylation, in particular, is required for adherence to human epithelial (HEp-2) cells, and involved in the immune stimulation of IL-8 production via TLR5 [27,28]. Similarly, in *A. hydrophila* AH-1, polar-flagella glycosylation has been shown to be involved in adherence, biofilm formation, and stimulation of the host immune response [29].

The glycosylation procedure generally entails the covalent attachment of glycans either to the amide nitrogen of Asn residues (*N*-glycosylation, first described in *C. jejuni* [53]), or to the hydroxyl oxygen of Ser, Thr or Tyr residues (*O*-glycosylation, described in all three domains of life [54]). In *Aeromonas* spp., only *O*-glycosylation has been reported to date, and it was first described in *A. caviae*. In this species, polar flagellins FlaA and FlaB were shown to be modified with pseudaminic acid (a nine-carbon nonulosonic sugar derivative of 373 Da, related to sialic acid) at 5–8 Ser or Thr residues of their central, immunogenic D2/D3 domains [43]. Additional *O*-glycosylated flagellins have later been described in other *Aeromonas* spp., with different glycosylation patterns. For instance, out of the one polar and two lateral flagellins described in *A. hydrophila* AH-1, only the polar flagellum has been shown to be glycosylated. As observed in *A. caviae* Sch3N, the glycan-modifying polar flagellin in *A. hydrophila* AH-1 has been shown to be a single monosaccharide, also speculated to be a pseudaminic-acid derivative [29]. On the contrary, in *A. piscicola* AH-3, which is known to have one polar and one lateral flagellin, both flagellar systems have been shown to be modified by the addition of sugars [25,55]. In this case, although the same pseudaminic-acid derivative is used to modify these structures, notable differences have been observed. For instance, *O*-glycosylation of the lateral flagellin LafA has been shown to occur at three flagellin sites, and always by the addition of a single pseudaminic-acid-derivative molecule of 376 Da. On the other hand, polar flagellins FlaA and FlaB have been shown to be glycosylated at one and six flagellin sites, respectively, and the pseudaminic-acid-derivative molecule can be found either alone or as part of a more complex glycan, composed of up to seven different sugar molecules. The longest form of this glycan (a 1679-Da heptasaccharide) is the only one identified in both *A. piscicola* AH-3 polar flagellins, and it is composed of two hexoses, three *N*-acetylhexosamines with a variable number of phosphate and methyl groups, one 102-Da monosaccharide, and the pseudaminic-acid-derivative molecule that links the glycan to the peptide [25]. Interestingly, in this species, a lipid carrier protein (WecX) has been shown to be involved in the *O*-glycosylation of both polar flagellins with this heptasaccharide [56].

Although the molecular mechanisms of *Aeromonas* spp. flagellin *O*-glycosylation are not fully understood, several genes involved in this process have been identified in the genomes of mesophilic species. In *A. piscicola* AH-3, genes involved in sugar biosynthesis are located near the polar-flagellin structural genes, and their mutation has been shown to affect both polar and lateral structures [55]. On the other hand, genes coding for glycosyltransferases (involved in the linkage of glycans to the polar or lateral flagellins) are found near the structural region of each flagellar system, and their mutation only affects one flagellar structure [39,40]. Interestingly, in *A. caviae* Sch3N, mutation of genes involved in pseudaminic-acid biosynthesis has been shown to affect both flagellar biogenesis and LPS O-antigen biosynthesis, suggesting a shared glycosylation route for these structures [43]. However, in *A. piscicola* AH-3, mutation of these genes only affects flagellar expression, implying that a different sugar and glycosylation pathway is used for LPS biosynthesis [55]. Among the genes coding for glycosyltransferases are the motility accessory factor (*maf*) genes, which have been identified in other Gram-negative bacteria that glycosylate their flagellins with nonulosonic acids (e.g., *H. pylori* and *C. jejuni*) [44,57]. In *Aeromonas* spp., particularly in *A. caviae* Sch3N, *maf*-*1* is suggested to be responsible for the transfer of the pseudaminic-acid derivative to the polar-flagellin monomers [26], and homologous genes have been found in *A. hydrophila* ATCC 7966T, *A. hydrophila* AH-1, and *A. piscicola* AH-3 [58,59,60]. A second motility accessory factor (*maf*-*2*), shown to be required for both polar- and lateral-flagella production in *A. piscicola* AH-3 [55], has been identified in *A. hydrophila* ATCC 7966T and some other mesophilic species [61], and yet another gene of this family (*maf*-*5*) has been shown to be required for lateral-flagella production in *A. piscicola* AH-3 [40]. Interestingly, *Aeromonas* spp. that modify polar flagellins with heterogeneous glycans (e.g., *A. piscicola* AH-3) present larger glycosylation islands in their genomes (containing several glycosyltransferase genes, including *maf*-*2*) than those that modify polar flagellins with a single pseudaminic-acid derivative (e.g., *A. hydrophila* AH-1 and *A. caviae* Sch3N) [61]. Moreover, a new glycosyltransferase gene (*fgi*-*1*) has been identified in the sequences of these species, as responsible for transferring the first sugar of the heterogeneous glycan to the pseudaminic-acid derivative that links the glycan to the polar flagellin.

## 3. Lipopolysaccharide

**Figure 2:**
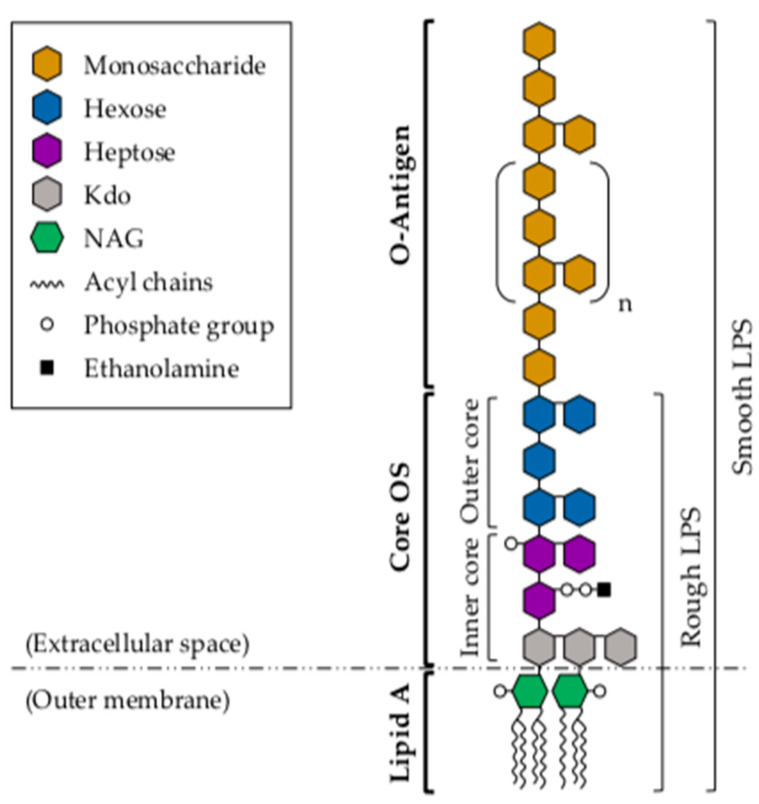
Schematic representation of the LPS molecule of Gram-negative bacteria. The lipid-A moiety consists of two parts: a lipid fraction composed of fatty-acid chains, which anchors LPS in the bacterial outer membrane, and a sugar backbone that links the molecule to the core oligosaccharide (core OS). The O-antigen chain, located at the most external part of the LPS molecule, is built of repetitive saccharide units that vary in number (n) among different bacterial cells. Smooth (S)-LPS is comprised of all three components, while rough (R)-LPS lacks the O-antigen subunit. The number and chemical structure of the acyl chains and sugar moieties represented in the figure can vary. NAG, *N*-acetylglucosamine. Kdo, 3-deoxy-d-*manno*-oct-2-ulosonic acid.

The surface glycoconjugate LPS, exclusively found in Gram-negative bacteria, is a major component of the bacterial outer membrane. LPS interaction with host immune cells triggers host inflammatory and immune responses through TLR4-mediated signaling, in association with myeloid differentiation protein 2 (MD-2) [30,62]. However, this primarily protective mechanism may become overshadowed by an acute pathophysiological response that leads to the overstimulation and release of proinflammatory cytokines, causing multiorgan dysfunction [31]. Structurally, LPS is comprised of three linked domains: a conserved and toxic lipid component, known as lipid A; the core oligosaccharide (core OS); and a highly variable O-specific-polysaccharide chain, known as the O-antigen (Figure 2) [63]. LPS molecules that contain all three regions are termed smooth (S)-LPS, while those lacking the O-antigen are referred to as rough (R)-LPS [64]. The biosynthesis of these structural components begins at the cytosolic membrane, in a process that involves a large number of enzymes and assembly proteins, encoded by more than 40 genes [65]. Once synthesized and assembled, the whole LPS molecule is subsequently transferred to the most external part of the bacterial outer membrane by the Lpt proteins, where it becomes surface-exposed [66].

### 3.1. Lipid A

The highly conserved lipid-A structure constitutes the hydrophobic, membrane-anchoring region of LPS, and plays an important role in the immunogenic properties of this molecule. For instance, it has been shown that the release of lipid A from lysed bacteria can provoke a major systemic inflammation, known as septic or endotoxic shock [67]. In addition, this molecule has been reported to induce B-cell polyclonal activation, which can lead to leukopenia, hemorrhagic necrosis of tumors, diarrhea, or even death [68].

Structurally, lipid A consists of a phosphorylated *N*-acetylglucosamine (NAG) dimer, with 6 or 7 saturated fatty acids attached to it (Figure 2) [67]. Some of these fatty acids are directly attached to the NAG dimer, while others have their terminal OH groups esterified with other fatty acids. The structural differences among lipid-A molecules are of particular importance, as the biological activity of LPS appears to depend on its specific conformation, which is in turn determined by the composition of each of its structural components. In *Aeromonas* spp., particularly in *A. salmonicida* subsp. *salmonicida*, three major molecules differing in their acylation patterns (tetra-, penta- or hexa-acylated) have been identified in the lipid-A structure [69]. The tetra-acylated molecule contains a 3-(dodecanoyloxy)tetradecanoic acid at N-2′, a 3-hydroxytetradecanoic acid at N-2, and a 3-hydroxytetradecanoic acid at O-3. The penta-acylated form has a similar fatty-acid-distribution pattern but, additionally, carries a 3-hydroxytetradecanoic acid at O-3′. In the hexa-acylated lipid-A structure, this 3-hydroxytetradecanoic acid molecule at O-3′ is esterified with a secondary 9-hexadecenoic acid, which has been associated with S-LPS. These findings are of significant importance, as the level of lipid-A acylation has been shown to be related to the host ability to recognize LPS in other Gram-negative species. In particular, reduced acylation of *Francisella novicida* lipid A results in reduced LPS recognition by murine caspase 11, but not by human caspases 4/5 [70,71]. This fact suggests that these lipid-A modifications could be critical for pathogenesis in certain mammals.

### 3.2. Core Oligosaccharide

The core OS is located between the lipid-A and O-antigen components of the LPS molecule (Figure 2). It is covalently attached to the lipid-A region, and it is therefore localized near the vicinity of the hydrophobic membrane. At the genetic level, three gene clusters have been associated with LPS core-OS biosynthesis in *Aeromonas* spp. [72,73,74,75], regions 2 and 3 being identical in *A. piscicola* AH-3 (O:34) and *A. salmonicida* strains A449 and A450. However, of the seven genes comprising region 1 in *A. salmonicida* A450, only three of them have been found to be identical in *A. piscicola* AH-3. Three other genes share high similarity between the two species, and the other one shows no homology whatsoever to any well-characterized gene [72,73]. In agreement with these observations, the comparison of the LPS core-OS structures of these two species renders a great similarity in the inner and part of the outer LPS core, while some differences can be observed in the distal part of the outer core. In *A. salmonicida*, the LPS core-OS has been shown to be composed of 8–12 sugars, and to be structurally divided into two regions with different sugar composition: the inner and the outer core [75]. The inner core is a highly phosphorylated region, and is therefore very anionic in nature. This part of the core is attached to the lipid-A molecule at the 6′ position of one NAG, and it contains 3-deoxy-d-*manno*-oct-2-ulosonic acid (Kdo), or the derivative residue 3-*glycero*-d-*talo*-oct-2-ulosonic acid [76]. Although the elemental structure of the inner core has been shown to typically contain l-*glycero*-d-mannoheptose (l,d-Hep), some *Aeromonas* spp. present D,D-Hep, either alone or in combination with l-d-Hep [77,78]. The inner core of *A. piscicola* AH-3 has been shown to be highly similar to that of *A. salmonicida* A449 [73,75], suggesting that it is a conserved structure within a genus or family. The outer core, on the other hand, is generally characterized by the presence of hexose sugars (e.g., glucose, galactose, *N*-acetyl galactosamine (GalNAc) and *N*-acetyl glucosamine (GlcNAc)) that provide an attachment site for the O-antigen structure. The comparison of the whole LPS core-OS structures of *A. salmonicida* A450 and *A. piscicola* AH-3 (O:34) shows that this region of the core presents more structural diversity than the inner core [72,73]. However, despite the differences reported between these two species, the whole LPS core-OS from *A. salmonicida* subsp. *pectinolytica* has been shown to be structurally identical to that of *A. piscicola* AH-3 (O:34), indicating that this outer core variation is still limited within a given species or genus [79].

### 3.3. O-Antigen

The O-antigen is the largest and most surface-exposed LPS component. It is usually attached to a terminal residue of the outer core, and it has been shown to mediate pathogenicity by protecting invading bacteria from the host immune response, particularly from the alternative complement cascade [80]. This structure constitutes the hydrophilic domain of the LPS molecule, and it is considered a major antigenic determinant of the Gram-negative cell wall. Structurally, O-antigens are composed of oligosaccharide polymers of various lengths, constituted by repeating subunits of 1–6 different sugars (Figure 2). The structural diversity of these O-polysaccharides (with more than 60 monosaccharides, different position and stereochemistry of the *O*-glycosidic bond, and presence or absence of different non-carbohydrate substituents) leads to great variability among Gram-negative species, and even strains [81]. This variability (specially at the terminal sugar) confers immunological specificity to the O-antigen, giving rise to a large number of O-antigen groups or serogroups. To date, 97 different serogroups have been identified in the *Aeromonas* genus, and there are still many strains not belonging to a known serogroup yet [82,83]. Of note, more than 60% of septicemia cases are related to serogroups O:11, O:16, O:18 and O:34 [84]. In particular, serogroup O:11 is associated with severe infections in humans (such as septicemia, meningitis and peritonitis), and serogroup O:34 (the most common among mesophilic *Aeromonas* spp.) is associated with wound infections in humans, and outbreaks of septicemia in fish.

In *Aeromonas* spp., LPS molecules are mainly highly heterogeneous mixtures of S-LPS with a varying proportion of ubiquitously located R-LPS. Given that the longer the sugar chains, the more hydrophilic the LPS molecule is, S- and R-LPS show marked dissimilarities in the kinetics of their blood clearance and cellular uptake [85]. More importantly, these molecules have a different ability to induce oxidative burst in human granulocytes, and to activate the host complement system [86]. Although it is known that both forms of LPS can signal through TLR4, only S-LPS requires the involvement of the CD14 antigen [87]. Therefore, since human neutrophils lack or express low amounts of this protein, only R-LPS is able to activate them through the canonical pathway [88]. In addition, in *A. piscicola* AH-3 (O:34), S-LPS has been shown to be prevalent at 20 °C (or 37 °C with high osmolarity), whereas R-LPS has been shown to be more common at 37 °C and low osmolarity [89,90]. This LPS thermoregulation has been linked to colonization, as cells grown at 20 °C show increased adherence to HEp-2 cells, and higher virulence in fish and mice than those grown at higher temperatures. In the same line, S-LPS has been shown to protect bacteria from the bactericide effects of the nonimmune serum, since the complement component C3b needs to bind to the long O-antigen chains far away from the membrane, and it is therefore unable to form the complement attack complex [91]. Despite the high variability of their chemical composition, some aeromonad O-antigen structures have been determined by chemical analysis of their genome sequences, or inferred by bioinformatics. Such is the case of the O-chain polysaccharides of *A. hydrophila* AH-1 (O:11), *A. piscicola* AH-3 (O:34), *A. salmonicida* subsp. *salmonicida*, *A. caviae* ATCC 15468, or *A. veronii* Bs19 (O:16) [74,77,92,93,94,95]. In addition, the genes involved in O-antigen biosynthesis have been described in *A. hydrophila* PPD134/91 (O:18) and *A. piscicola* AH-3 (O:34) [96,97]. As reported for other clusters involved in polysaccharide biosynthesis, three classes of genes have been found: those that code for glycosyltransferases, those involved in the biosynthesis of activated sugars, and those whose products are necessary for O-antigen translocation and polymerization. Of particular note, sequence comparison of different *Aeromonas* spp. has shown epidemic strains to have larger O-antigen gene clusters than those previously reported for *A. piscicola* AH-3 and *A. hydrophila* PPD134/91 [98]. Moreover, genes for 3-acetamido-3,6-dideoxy-d-galactose biosynthesis are present in all the disease-causing strains but not in the reference or previously sequenced ones, suggesting an important role of this sugar in the O-antigen structure of epidemic strains.

On a different aspect, it is known that the O-antigen is the last structural part added to the LPS molecule, before the whole complex is exported to the external side of the outer membrane by the Lpt proteins [66]. However, prior to its attachment to the lipid-A-core-OS structure of LPS (with the help of the WaaL ligase [99]), the O-antigen needs to be fully synthesized. O-antigen biosynthesis begins with the generation of a lipid-linked glycan intermediate, in a process similar to the biogenesis of lipid-linked oligosaccharides for protein *N*-glycosylation [100]. Whereas eukaryotic and archaeal cells use dolichyl phosphate, this lipid-linked glycan intermediate is, in bacteria, undecaprenyl phosphate (UndP). Once generated, a sugar transfer reaction takes place, in a process involving a membrane-associated polyprenol phosphate acceptor and a cytoplasmic UDP-D-*N*-acetylhexosamine sugar donor. Although some strains are known to use the WecA enzyme to transfer *N*-acetyl-glucosamine-1-phosphate [101], *A. piscicola* AH-3 has been shown to use WecP to transfer *N*-acetyl-galactosamine instead [102], denoting a different phylogenetical branch. Following this reaction, the O-antigen unit is flipped across the bacterial inner membrane by the Wzx protein [103], and assembled on the periplasmic side of the inner membrane by the Wzy O-antigen polymerase [104]. The O-antigen is elongated until it reaches the final polymer length, in a process regulated by the O-antigen chain length regulator Wzz [105]. This has been shown to occur through the generation of glycosidic bonds between the non-reducing end of a new UndPP-linked O-antigen unit and the reducing end of the UndPP-linked growing polymer [106]. Of the four assembly pathways identified to date (the Wzx/Wzy- and Wzm/Wzt-dependent schemes being the most prevalent ones [107]), *A. piscicola* AH-3 (O:34) has been shown to follow the Wzx/Wzy-dependent route, as both *wzy* and *wzx* genes are found in the O-antigen gene cluster of this strain [97]. Interestingly, and in correlation with the previously mentioned findings on LPS thermoregulation, the gene that codes for Wzz has a much higher transcription at 20 °C than at 37 °C. In addition, although the first sugar of the O-antigen repeating unit seems to be determinant for the generation of glycosidic bonds [108], the Wzy enzyme of *A. piscicola* AH-3 (O:34) has been shown to be permissive with this first sugar at the non-reducing end [109].

## 4. Capsular Polysaccharide

Although *Aeromonas* spp. have historically been described as non-encapsulated bacteria, several CPSs have been identified in both motile and non-motile species, and the chemical composition of some of these capsules has actually been described (Table 1). Mesophilic *Aeromonas* spp. belonging to serotypes O:11 (e.g., *A. hydrophila* TF7 and LL1) and O:34 (e.g., *A. hydrophila* Ba5 and *A. piscicola* AH3) have been shown to produce a capsule composed of d-glucose, d-mannose, l-rhamnose, d-mannuronic acid, and acetic acid heteropolymers (in distinct molar ratios, determined by the species serogroup) [32]. Regarding non-motile *Aeromonas* spp., *A. salmonicida* A449, A450 and A894 have been shown to produce a similar capsule, composed of d-glucose, d-mannose, l-rhamnose, *N*-acetylmannosamine, and mannuronic acid [33]. However, *A. salmonicida* 80204-1 produces a rather different CPS, composed of repeating units of a linear trisaccharide of 2-acetamido-2-deoxy-d-quinovose, 3-[(*N*-acetyl-L-alanyl) amido]-3-deoxy-d-quinovose, and 2-acetamido-2-deoxy-d-galacturonic acid [114]. Interestingly, in this species, the capsular composition is identical to that of the LPS O-antigen, which is a feature also reported in other fish pathogens [115,116]. Another capsule of different chemical composition has been recently identified in the *Aeromonas* sp. strain AMG272, isolated from agricultural soil. In this case, the CPS is a large heteropolysaccharide, composed of repeating units of a branched pentasaccharide of d-galactose, *N*-acetyl-d-glucosamine, *N*-acetyl-d-galactosamine, and 3-acetamido-4-*O*-acetyl-3,6-dideoxy-d-galactose [117].

The bacterial capsule is a highly hydrated structure found on the cell surface of a broad range of bacterial species. It is composed of large polysaccharides, usually negatively charged, that extend far beyond the cell-wall components to produce a thick, protective coat around the entire bacterial cell. CPSs can sometimes be associated with the bacterial cell in the absence of a membrane anchor, but are usually found covalently attached to other cell-surface molecules, such as phospholipid or lipid A [63]. These structures are composed of repeating monosaccharide units that are joined together by glycosidic bonds, forming homo- or heteropolymers. Given that all hydroxyl groups present within each monosaccharide may be involved in the formation of a glycosidic bond, the union between any two monosaccharides constituting the polysaccharide chain can occur in numerous configurations, leading to a large structural diversity among bacterial capsules [110,111]. Despite this great diversity, chemically identical capsular polysaccharides may also be synthesized by different bacterial species. For instance, the *E. coli* K1 antigen (a homopolymer of α-2,8-*N*-acetylneuraminic acid, shown to be the major cause of neonatal meningitis [112]), has been shown to be identical to the *Neisseria meningitidis* group B capsule [113].

At the genetic level, genes associated with capsular biosynthesis and export are shown to be distributed at a single chromosomal locus, and their arrangement seems to be conserved in most bacterial species [111]. In *E. coli*, capsules have been classified into four groups, based on their genetic and biosynthetic organization [118,119]. While capsules of groups 1 and 4 are assembled and exported via the Wzy-dependent pathway, those of groups 2 and 3 use the ATP-dependent pathway, and are in turn organized into 3 regions. Genes of regions 1 and 3 are involved in the export and modification of CPSs, and have been shown to be conserved within a strain. Region 2, on the other hand, contains genes responsible for CPS biosynthesis, and is usually serotype-specific [118]. Although these regions are generally organized into one transcriptional unit, some genes within a region may be translationally coupled, allowing for the balanced expression of two different proteins. Such is the case of group-2 genes *kpsU* and *kpsC*, and group-3 genes *kpsM* and *kpsT* [111]. In *Aeromonas* spp., the genes required for CPS biosynthesis and export were first described in *A. hydrophila* PPD134/91 and JCM3980 (O:18) [96,120]. In these species, the capsule clusters contain 13 genes and, as observed in *E. coli*, are arranged into three distinct regions. Again, genes of regions 1 and 3 are involved in capsule maturation and export [96], while those of region 2 are responsible for CPS biosynthesis, and are serotype-specific. Interestingly, two different capsules have been identified in *Aeromonas* spp. region 2: 2A and 2B. The gene cluster of 2A capsules (mainly found in serogroups O:18 and O:34) is about 10 kb long and contains five ORFs, while that of 2B capsules (found in serogroups O:21 and O:27) is about 5 kb long and contains four ORFs [120]. Of particular note, although no clusters involved in capsule biosynthesis have been identified in *A. hydrophila* 1051-88 (O:34), two genes (*orf1* and *wcaJ*) have been described as responsible for CPS production in this strain [121].

Since bacterial capsules often constitute the outermost layer of the cell, they are frequently involved in mediating direct interactions between bacteria and their environment. It is due to these interactions that CPSs are considered important virulence factors for many bacterial pathogens. In fact, several functions have been assigned to these surface structures, including prevention of desiccation, adherence, biofilm formation, resistance to both specific and non-specific host immunity, and mediating the diffusion of molecules through the cell surface [110]. In *Aeromonas* spp., several studies have demonstrated that CPSs contribute to pathogenesis in vivo, and have a critical role in host–pathogen interactions. For instance, *A. salmonicida* has been shown to respond to the hostile intraperitoneal environment of rainbow trout by inducing the synthesis of a capsule, which has been directly related to bacterial resistance against host lytic factors and phagocytosis, and seems to protect *A. salmonicida* from the host complement system [122]. Moreover, capsule biosynthesis has also been shown to contribute to bacterial adhesion to different fish cell lines in *A. salmonicida* and *A. piscicola*, increasing the invasion and survival abilities of these species [123,124]. In particular, purified group-2 capsules of the *A. hydrophila* virulent strain PPD134/91 have been shown to confer resistance to serum-mediated killing in the avirulent strain PPD35/85 [96].

## 5. α-Glucan

**Figure 3:**
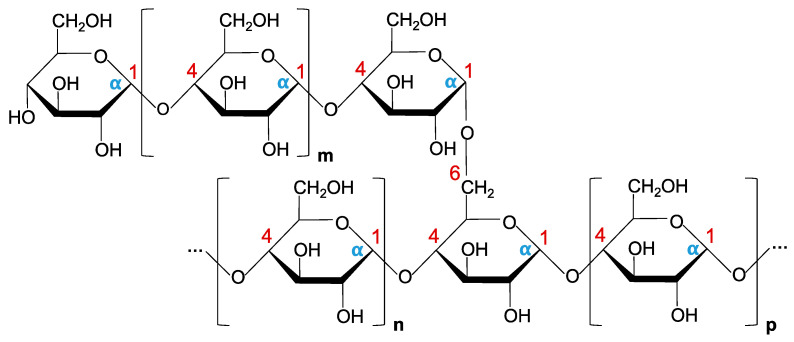
Structure representation of the surface α-glucan described in *A*. *piscicola* AH-3. The polysaccharide chain is comprised of α-d-glucosyl units connected by α-1,4-linkages, and branched through α-1,6-glycosidic bonds (m, n, and p stand for different numbers of repeated d-glucose monomers).

Glucans are the most widespread polysaccharides in nature. They are composed of d-glucose monomers linked to each other by glycosidic bonds, and show a great chemical and structural diversity. According to their polymer conformation, bacterial glucans are divided into two major types: α-glucan and β-glucan. Out of the several different α-glucans described in Gram-negative bacteria, glycogen is the most studied one. This biopolymer serves as the major carbon- and energy-storage compound, and is thus typically accumulated under nutrient-depletion conditions [125]. Structurally, glycogen is known to be comprised of α-d-glucosyl units connected by α-1,4-linkages, and branched through α-1,6-glycosidic bonds [126]. Depending on the source, glycogen molecules vary in chain length and branching frequency, which determines their rate of degradation, long and highly branched chains being more rapidly degraded than shorter and slightly branched ones [127]. In *E. coli*, glucose-1-phosphate has been shown to be the early precursor for glycogen synthesis. This molecule is first converted to ADP-glucose (ADP-Glc), with the aid of the ADP-Glc pyrophosphorylase GlgC. These ADP-Glc units are then transferred to the nonreducing end of the α-1,4-glucan chain by the glycogen synthase GlgA, or branched in α-1,6 linkages by a branching enzyme [125,126]. In *Aeromonas* spp., a similar surface α-glucan has been described in *A. piscicola* AH-3, and *A. hydrophila* strains AH-1 and PPD134/91 [34]. This glucan, highly expressed at temperatures below 20 °C, is also comprised of α-d-glucosyl units connected by α-1,4-linkages, and branched through α-1,6-glycosidic bonds (Figure 3). It is synthesized via UDP-Glc (instead of ADP-Glc, as described in *E. coli*), with the help of the UDP-Glc pyrophosphorylase GlgC (as opposed to ADP-Glc pyrophosphorylase GlgC) and the glycogen synthase GlgA. Interestingly, while *E. coli* GlgA reacts exclusively with ADP-Glc, *Aeromonas* spp. GlgA is able to use UDP-Glc to produce α-glucan, and probably ADP-Glc as well for glycogen biosynthesis. In addition, in *A. piscicola* AH-3, the absence of GlgC does not seem to affect either LPS O-antigen or α-glucan biosynthesis, while the absence of GlgA results in incorrect LPS core-OS formation and reduced α-glucan production. Moreover, *A. piscicola* AH-3 synthesizes UDP-Glc, also needed for the formation of the LPS inner core, via both GlgC and GalU. In the absence of GalU (which consequently leads to reduced levels of UDP-Glc), *A. piscicola* AH-3 establishes a preference for survival and pathogenesis, abolishing the formation of surface α-glucan and rather producing a complete LPS core. Surprisingly, GalU mutants have been shown to lack the LPS O-antigen fraction, although this is suggested to occur because of their inability to incorporate the terminal galactose residue to the LPS core-OS structure [128]. Of particular note, in this species, surface α-glucan and LPS O-antigen are both exported via WecP, and ligated to the bacterial surface through WaaL, despite being independent polysaccharides [34].

On a different aspect, several studies have revealed that bacterial exopolysaccharides are essential for biofilm formation [34,129,130]. In this regard, the role of *Aeromonas* spp. α-glucan could be similar to that of some *E. coli* exopolysaccharides (e.g., colanic acid and poly-β-1,6-*N*-acetyl-d-glucosamine (PNAG) [131]), which are integral elements of biofilms and hold together the different protein, lipid and polysaccharide components of these layers [130]. In addition, *Aeromonas* spp. α-glucan may play a role in modulating the host immune response, as it has been suggested in other bacterial species such as *Mycobacterium tuberculosis* [132]. Unlike the LPS O-antigen, which has a predominant role in both cell adhesion and biofilm formation, α-glucan does not seem to have a significant role in *Aeromonas* spp. cell adhesion, which supports the preference of these bacteria to produce LPS rather than α-glucan [34].

## 6. Future Perspectives and Concluding Remarks

Among the different pathogenic factors associated with *Aeromonas* spp. virulence, surface glucans have been shown to play an important role in host–pathogen interactions, contributing to adherence, colonization, and overall survival of pathogenic strains. Given that the number of reported infections caused by *Aeromonas* spp. has been steadily rising in recent years [13], fully understanding the mechanisms underlying the biogenesis and regulation of *Aeromonas* spp. surface glucan structures seems to be crucial for novel therapeutic strategies. In this regard, some advances have been made in recent years. LPS, for instance, has been shown to be a key elicitor of the host immune system [30], but also to provoke an acute pathophysiological response that causes damage to tissues and organs [31]. To overcome this problem, LPS variants that stimulate the immune response without toxic effects have been explored as immunotherapeutics. For instance, outer membrane vesicles containing LPS molecules with their core oligosaccharide modified with specific pathogen-associated glycans (e.g., capsule or heterologous O-antigens) have been shown to provide protection against the associated pathogen [133,134,135]. Similarly, *Salmonella minnesota* LPS with chemical variations in its lipid-A molecule has been used as a vaccine adjuvant in mice, resulting in reduced activation of the MyD88-dependent response [136]. By overexpressing and knocking out genes involved in lipid-A synthesis and modification, other studies have also bioengineered various Gram-negative species (e.g., *Yersinia pestis, Neisseria meningitidis*, and non-pathogenic *E. coli* strains) to produce different LPS glycoforms [137,138,139]. Although a similar range of immune stimulation was obtained with all bioengineered species, not all trends observed for the immune recognition of *E. coli* lipid A held true for *N. meningitidis*. This fact highlights the importance of specifically studying the LPS-dependent immune modulation caused by *Aeromonas* spp. In the same line, bacterial capsules have been historically used as vaccine target antigens for some bacterial species [140,141], although the emergence of antibiotic-resistant bacteria in recent years makes it necessary to find alternative approaches to combat this public health crisis. In this regard, it will be critical to identify the signals that stimulate and/or repress CPS biosynthesis in *Aeromonas* spp., and unravel the detailed network of molecular receptors and effectors of such signals. Given that flagellin glycosylation seems to be involved in several biological functions related to *Aeromonas* spp. pathogenesis and virulence [27,28,29], fully understanding the molecular mechanisms underlying such process may also allow for the development of novel antimicrobial strategies. Similarly, in regard to α-glucan, several dendritic cells and monocyte receptors have been related to the recognition of this surface structure in *Mycobacterium tuberculosis* [132], and it would not be surprising that the *Aeromonas* spp. α-glucan could be involved in a similar role, modulating the host immune response. Given that it also seems to play a fundamental role in biofilm formation and overall integrity of the bacterial cell envelope [130], understanding the mechanisms that regulate α-glucan biosynthesis could be of critical importance for improving our current strategies against *Aeromonas* spp. disease. For instance, several published reports indicate that the use of yeast β-glucans increases fish resistance to *Aeromonas* spp. infection, by enhancing the non-specific immune response [142,143]. In this regard, administration of *Aeromonas* spp. α-glucan, instead of yeast β-glucans, could represent an improved strategy for providing protection against *Aeromonas* spp. disease.

## Figures and Tables

**Table 1:** Described chemical compositions of *Aeromonas* spp. capsular polysaccharides. The different capsules are listed in chronological order, as they have been determined. Glc, glucose. Man, mannose. Rha, rhamnose. ManA, mannuronic acid. ManNAc, *N*-acetylmannosamine. QuiNAc, 2-acetamido-2-deoxy-d-quinovose. Qui3NAlaNAc, 3-[(*N*-acetyl-L-alanyl) amido]-3-deoxy-d-quinovose. GalNAcA, 2-acetamido-2-deoxy-d-galacturonic acid. Gal, galactose. GlcNAc, *N*-acetyl-d-glucosamine. GalNAc, *N*-acetyl-d-galactosamine. Fuc3Nac4Ac, 3-acetamido-4-*O*-acetyl-3,6-dideoxy-d-galactose.

Capsule Composition	Species (Strains)	Ref.
D-Glc, D-Man, L-Rha, ManNAc, D-ManA	*A. salmonicida* (A449, A450, A894)	[33]
D-Glc, D-Man, L-Rha, D-ManA, Acetic acid	*A. hydrophila* (TF7, LL1, Ba5)	[32]
	*A. piscicola* (AH3)	[32]
QuiNAc, Qui3NAlaNAc, GalNAcA	*A. salmonicida* (80204-1)	[114]
D-Gal, GlcNAc, GalNAc, D-Fuc3NAc4Ac	*Aeromonas* sp. (AMG272)	[117]
